# A survey of physician experience and treatment satisfaction prescribing once-weekly semaglutide injections for patients with type 2 diabetes in Canada

**DOI:** 10.1097/XCE.0000000000000260

**Published:** 2022-03-23

**Authors:** Kamran Qureshy, Andreas Ross Kirk, Michael Lyng Wolden, Amir Abbas Mohseni Zonoozi, Aiden Liu

**Affiliations:** a Complete Endocrine Care, Vaughan, Ontario, Canada; b Novo Nordisk A/S, Søborg, Denmark; c Novo Nordisk Canada Inc., Mississauga, Ontario, Canada

**Keywords:** injection, once-weekly, physician experience, semaglutide, survey, treatment satisfaction, type 2 diabetes

## Abstract

We assessed physicians’ experiences of prescribing once-weekly (OW) semaglutide to patients with type 2 diabetes (T2D) in Canada. Physicians who had prescribed OW semaglutide to ≥2 patients with T2D in the past 12 months and had been doing so for ≥3 months were surveyed during 1–17 October 2018. Prescribing reasons, treatment satisfaction and reasons for discontinuation were assessed. Of the 50 participants, 72% and 54% were prescribed OW semaglutide due to its superior glycemic control and effect on weight, respectively. Most physicians were more satisfied with injection frequency (62%), effect on weight (60%), achieving HbA1c target (54%) and therapy simplicity (50%) with OW semaglutide versus other glucagon-like peptide-1 receptor agonists. Treatment discontinuations in 13% of OW semaglutide-treated patients were reported by physicians, primarily due to gastrointestinal symptoms (70%). The survey suggests that physicians are satisfied with the OW semaglutide clinical effects.

Video Abstract: http://links.lww.com/CAEN/A34

## Introduction

Once-weekly (OW) subcutaneous semaglutide is a glucagon-like peptide-1 receptor agonist (GLP-1 RA) approved in Canada on 4 January 2018 for treating adults with type 2 diabetes (T2D) [1]. OW semaglutide was proven to be a safe and efficacious intervention across multiple patient populations with T2D, as monotherapy and combined with oral antihyperglycemic drugs (OADs) or insulin [2–8].

The Diabetes Canada pharmacotherapy guidelines recommend GLP-1 RAs as add-on to metformin if glycemic target cannot be attained with metformin alone [9]. This survey studied the characteristics of patients initiating OW semaglutide and the characteristics, perception, behavior and experiences of physicians who have prescribed OW semaglutide to patients with T2D in Canada.

## Methods

### Survey design and participants

A structured online survey for primary care practitioners (PCPs) and specialists treating patients with T2D was conducted from 1–17 October 2018 in Canada (end of the survey); the target sample size of 50 physicians was met. Eligible physicians included those who had prescribed OW semaglutide to at least two patients with T2D in the past 12 months and had been doing so for at least 3 months.

### Informed consent process, confidentiality and data protection

Participants consented to have their contact details forwarded to the sponsor for reporting of adverse effects. Personal information and responses of participants were kept confidential, and applicable data protection laws were followed.

### Survey questionnaire and administration

The survey questionnaire extracted information on characteristics of prescribing physicians and patients who were prescribed OW semaglutide, prescribing reasons, treatment response, physicians’ satisfaction, reasons for discontinuation, and physician and patient concerns with OW semaglutide, compared with other GLP-1 RAs (Supplementary Material, Supplemental Digital Content 2, http://links.lww.com/CAEN/A35).

Physicians were asked to rank statements from one to five regarding reasons to prescribe OW semaglutide (rank 1–2 = agree; rank 3 = neither agree nor disagree; rank 4–5 = disagree) and characteristics of patients receiving OW semaglutide. Physicians ranked statements regarding satisfaction level in five increments, from ‘much more satisfied with OW semaglutide than other GLP-1 RAs’ to ‘much more satisfied with other GLP-1 RAs than OW semaglutide’, or ‘don’t know’.

### Analysis

All data were based on the fully completed surveys and analyzed using a *t*-test at a 90% confidence limit.

## Results

### Characteristics of physicians who prescribed and patients who were prescribed once-weekly semaglutide

On average, for each physician, 546 patients with T2D were seen in the past 12 months, and 42 were treated with OW semaglutide (overall 50 participants, Table 1). Nearly half (48%) of physicians had at least 4 months of experience prescribing OW semaglutide. Of the patients prescribed OW semaglutide, 94% had inadequately controlled blood glucose, and 78% were overweight/obese (Table 2).

**Table 1.** Characteristics of 50 surveyed physicians prescribing once-weekly semaglutide to patients with type 2 diabetes in Canada

	Total	PCPs	Specialists
	50 (100)	25 (100)	25 (100)
Primary specialty			
General/family practice	23 (46)	23 (92)	ND
Internal medicine	2 (4)	2 (8)	ND
Cardiology	9 (18)	ND	9 (36)
Endocrinology	13 (26)	ND	13 (52)
Diabetology	3 (6)	ND	3 (12)
Time since first prescription of OW semaglutide			
3 months	16 (32)	7 (28)	9 (36)
3 to ≤4 months	10 (20)	6 (24)	4 (16)
4 to ≤5 months	5 (10)	2 (8)	3 (12)
5 to ≤6 months	14 (28)	6 (24)	8 (32)
6 to ≤7 months	5 (10)	4 (16)	1 (4)
Time since last prescription of OW semaglutide			
<14 days	35 (70)	19 (76)	16 (64)
15 days to ≤1 month	9 (18)	5 (20)	4 (16)
1 to ≤2 months	5 (10)	1 (4)	4 (16)
2 to ≤3 months	0 (0)	0 (0)	0 (0)
3 to ≤4 months	1 (2)	0 (0)	1 (4)
Average number of patients with T2D seen per responder in the last 12 months	546 (100)	385 (100)	707 (100)
Patients with T2D treated with OW semaglutide per responder	42 (8)	39 (10)	45 (6)

Data presented are *n* (%) unless otherwise stated.

OW, once-weekly; ND, no data; PCPs, primary care practitioners; T2D, type 2 diabetes.

**Table 2.** Characteristics of patients with type 2 diabetes at once-weekly semaglutide initiation, as reported by 50 prescribing physicians in Canada

	Total	PCPs	Specialists
Baseline treatment regimen, %			
1 OAD	26	27	24
2 OADs^a^	28	36	19
≥3 OADs	14	11	18
GLP-1 RA ± OAD	7	7	8
Basal ± OAD	12	9	14
Basal + bolus ± OAD^a^	7	4	10
Basal + GLP-1 RA ± OAD	5	4	5
CSII/insulin pump	0	0	0
Treatment-naïve	1	1	2
Age, %			
<18 years	1	1	1
18–25 years	7	5	9
36–50 years	33	34	33
51–65 years	40	43	36
>65 years	19	17	21
BMI, %			
<30 kg/m^2^	13	12	14
30–35 kg/m^2^	46	47	46
>35 kg/m^2^	38	40	36
Unknown	3	2	5
HbA1c, %			
<7.0% (53 mmol/mol)	4	3	5
7.0–7.5% (53–59 mmol/mol)	14	15	14
7.5–8.0% (59–64 mmol/mol)	24	23	25
8.0–8.5% (64–69 mmol/mol)	27	29	25
8.5–9.0% (69–75 mmol/mol)	18	20	17
>9.0% (75 mmol/mol)	12	10	15
Patients with inadequately controlled blood glucose (HbA1c), %	94	92	96
Patients with excess weight, %	78	88	68
Patients who find adherence to previous treatment difficult, %	60	64	56
Patients with established CV risk, such as previous stroke or MI^a^, %	56	76	36
Patients with risk of hypoglycemia, %	46	52	40

Percentage values may not total 100 due to rounding.

CSII, continuous subcutaneous insulin infusion; CV, cardiovascular; GLP-1 RA, glucagon-like peptide-1 receptor agonist; HbA1c, glycated hemoglobin; MI, myocardial infarction; *n*, number of patients; OADs, oral antihyperglyemic drugs; OW, once-weekly; PCPs, primary care practitioners; T2D, type 2 diabetes.

^a^Significant difference between PCPs and specialists at the 90% confidence limit.

### Reasons for prescribing once-weekly semaglutide to patients with type 2 diabetes

**Fig. 1.**
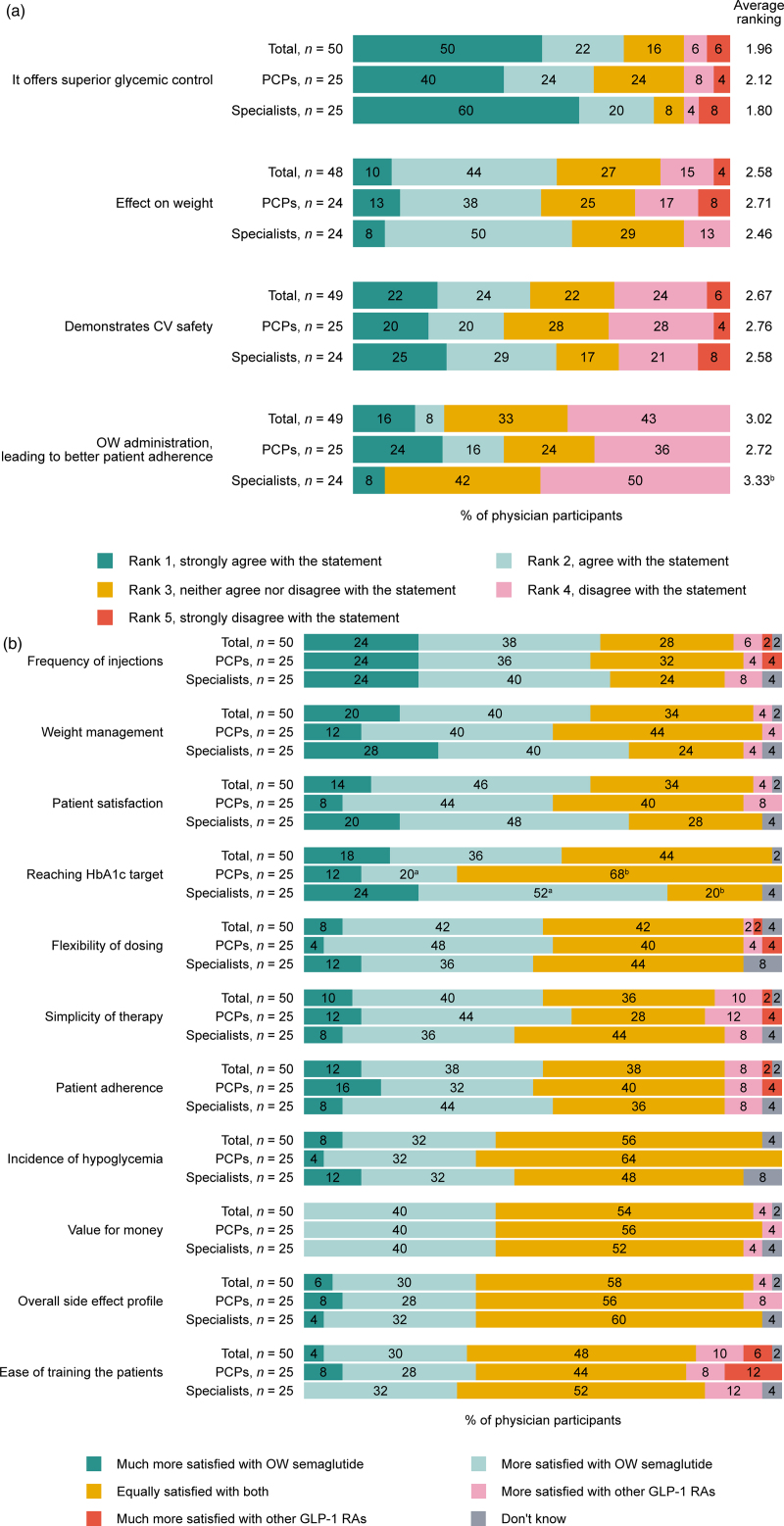
(a) Reasons for prescribing OW semaglutide for patients with T2D^a^, as reported by 50 prescribing physicians in Canada. (b) Physicians’ satisfaction level with OW semaglutide compared with other GLP-1 RAs for patients with T2D. (a) Percentage values may not total 100 due to rounding. Question: What are your main reasons for prescribing Ozempic (semaglutide) to your patients with T2D? ^a^Other reasons included: it provides immediate results and better device option, it offers value for money and it is not applicable. ^b^The average ranking from specialists was significantly higher than that from the PCPs at 90% confidence limit. CV, cardiovascular; *n*, number of patients; OW, once-weekly; PCPs, primary care practitioners; T2D, type 2 diabetes. (b) Question: please compare your level of satisfaction when treating patients with Ozempic (semaglutide) compared with other GLP-1 RAs based on each of the following. ^a^Significantly more specialists were satisfied with OW semaglutide compared with PCPs at 90% confidence limit. ^b^Significantly more PCPs were equality satisfied with OW semaglutide and other GLP-1 RAs, compared with specialists at 90% confidence limit. GLP-1 RA, glucagon-like peptide-1 receptor agonist; HbA1c, glycated hemoglobin; OW, once-weekly; *n*, number of participants; PCP, primary care practitioner; T2D, type 2 diabetes.

Of all the participating physicians, 72% and 54% cited superior glycemic control versus other GLP-1 RAs and effect on weight as their reasons for prescribing OW semaglutide, respectively (Fig. 1a). Fewer physicians cited cardiovascular (CV) safety (46%) and OW administration (24%) as their reasons for prescribing OW semaglutide (Fig. 1a).

### Treatment response

Physicians prescribed OW semaglutide alone, OW semaglutide + OADs and OW semaglutide + insulin to 12%, 69% and 19% of their patients, respectively. Following OW semaglutide initiation, physicians reported that 65% of their patients had reached their glycemic target, and 55% of patients’ HbA1c was lower than 7% (Supplementary Material, Fig. S1, Supplemental Digital Content 2, http://links.lww.com/CAEN/A35). Furthermore, PCPs were more inclined to use the 0.5-mg maintenance dose of OW semaglutide, whereas specialists were inclined to use the 1-mg maintenance dose (Fig. S2, Supplemental Digital Content 2, http://links.lww.com/CAEN/A35).

### Physicians’ satisfaction compared with other glucagon-like peptide-1 receptor agonists

Most physicians were more satisfied with frequency of injections (62%), weight management (60%), achieving HbA1c target (54%) and simplicity of therapy (50%) with OW semaglutide versus other GLP-1 RAs (Fig. 1b).

### Discontinuation of once-weekly semaglutide

According to physicians, ~13% of their patients who initiated OW semaglutide discontinued the treatment due to gastrointestinal (GI) side effects (70%), treatment affordability (69%), insufficient weight loss (39%) and desired blood glucose control not fully achieved (39%) (Supplementary Material, Fig. S3, Supplemental Digital Content 2, http://links.lww.com/CAEN/A35). Of the patients who discontinued the treatment, 43% were reported to have done so within the first month and 78% within the first 3 months of initiation.

### Physician and patient concerns with once-weekly semaglutide

According to the participating physicians, the most common patient concerns with OW semaglutide were treatment affordability and GI side effects (Supplementary Material, Fig. S4, Supplemental Digital Content 2, http://links.lww.com/CAEN/A35). When asked to compare these concerns with OW semaglutide versus other GLP-1 RAs, 98% and 92% of physicians, respectively, stated they were either equally or less concerned with OW semaglutide. Regarding patients’ confidence in treatments, 68% of physicians reported that patients felt more motivated to reach their glycemic target, and an additional 30% of physicians reported patients felt equally motivated to reach their glycemic target with OW semaglutide versus other GLP-1 RAs.

## Discussion

Results in this survey provide insights into the real-world experiences of physicians who prescribed OW semaglutide to patients with T2D in Canada.

Most physicians prescribed OW semaglutide due to its superior glycemic control and weight loss benefit, consistent with previous studies [2,3,5–8,10,11]. CV safety was the third most popular reason, probably because at the time of the survey, no CV benefit of OW semaglutide was cited by any regulatory labels [12–14]. However, current guidelines are shifting focus to the CV benefit of glucose-lowering medications [15–17], and the CV safety/benefit of OW semaglutide has been included [9,17].

Top reasons for treatment discontinuation cited by the physicians were GI side effects and treatment affordability, consistent with the safety of OW semaglutide [2–5,7,8] and previous literature [18,19].

Of note, at the time of the survey, OW semaglutide was not publicly reimbursed in Canada and was only recommended to be reimbursed in 2019 [20]. Given the cost-effectiveness of OW semaglutide [21–28], it may represent a more cost-effective treatment option versus other GLP-1 RAs. Moreover, given the timing of the survey in 2018, when OW semaglutide was new to the market, our report likely provides conservative opinions. Physician experience in 2022 may be increasingly positive, as OW semaglutide is more widely prescribed [29,30] and recommended as a second-line agent in the Diabetes Canada Guidelines [17].

One limitation is that the findings are heavily based on a small number of physicians’ experiences.

## Conclusion

In this survey, most physicians prescribed OW semaglutide due to its superior glycemic effects and effect on weight, and were more satisfied with OW semaglutide versus other GLP-1 RAs. Participating physicians believed that patients with T2D taking OW semaglutide were more satisfied with the frequency of injections and more confident in reaching their glycemic target versus other GLP-1 RAs.

## Acknowledgements

Writing and editorial support was provided by Jin Heppell, PhD, and Izabel James, MBBS, of Ashfield MedComms, an Ashfield Health company, funded by Novo Nordisk A/S (Søborg, Denmark). This study and the journal’s Rapid Service were sponsored by Novo Nordisk A/S (Søborg, Denmark).

Part of the manuscript has been previously presented at the Diabetes Canada/Canadian Society of Endocrinology and Metabolism Professional Conference, 2–5 October 2019, Winnipeg, MB, Canada.

Participating physicians provided their consent to participate in the study and waived confidentiality so that their names/contact details could be forwarded to the sponsor’s pharmacovigilance department in the event of reported adverse effects. However, no mechanisms were in place to associate names of the physicians with responses given. Personal information of participants were anonymized, and responses remained confidential. Participating physicians were informed that data would be stored securely on IQVIA servers, in accordance with applicable data protection laws, only for as long as necessary, for the purposes of use outlined in study agreement. The study was performed in accordance with the General Data Protection Regulation and Novo Nordisk A/S adverse events and compliance requirements.

The datasets generated during and/or analyzed during the current study are available from the corresponding author on reasonable request.

### Conflicts of interest

K.Q.: honoraria and/or grant support from Novo Nordisk, Merck, Eli Lily, BI, AstraZeneca and Abbot. A.A.M.Z. and A.L.: employees of Novo Nordisk Canada Inc. A.R.K. and M.L.W.: employees and shareholders of Novo Nordisk A/S.

## Supplementary Material


