# Supplementary material for: A survey of physician experience and treatment satisfaction prescribing once-weekly semaglutide injections for patients with type 2 diabetes in Canada
Source: Cardiovasc Endocrinol Metab. 2022 Mar 23;11(2):e0260. doi: 10.1097/XCE.0000000000000260 (PMC8955057; doi:10.1097/XCE.0000000000000260)
Supplement: Supplementary file 2 [file xce-11-e0260-s002.pdf]

Supplementary material

A Survey of Physician Experience and Treatment Satisfaction Prescribing Once-Weekly Semaglutide Injections for Patients with Type 2 Diabetes in Canada

Kamran Qureshy[1], Andreas Ross Kirk[2], Michael Lyng Wolden[2], Amir Abbas Mohseni Zonoozi[3], Aiden Liu[3]

[1]Complete Endocrine Care, Vaughan, ON, Canada

[2]Novo Nordisk A/S, Søborg, Denmark

[3]Novo Nordisk Canada Inc., Mississauga, ON, Canada

Contents:

- Fig. S1: Proportions of patients with T2D treated with OW semaglutide who reached and did not reach their HbA1c target, as reported by 50 prescribing physicians in Canada
- Fig. S2: Proportions of patients with T2D who reached and did not reach their HbA1c target receiving 0.5 mg or 1.0 mg OW semaglutide, as reported by 50 prescribing physicians in Canada
- Fig. S3: Physician-reported reasons for discontinuation of OW semaglutide[a] in patients with T2D in Canada
- Fig. S4: Physician-reported patient concerns with OW semaglutide for T2D in Canada
- Ozempic® (semaglutide) Physician Understanding and Experience Survey

Fig. S1: Proportions of patients with T2D treated with OW semaglutide who reached and did not reach their HbA1c target, as reported by 50 prescribing physicians in Canada

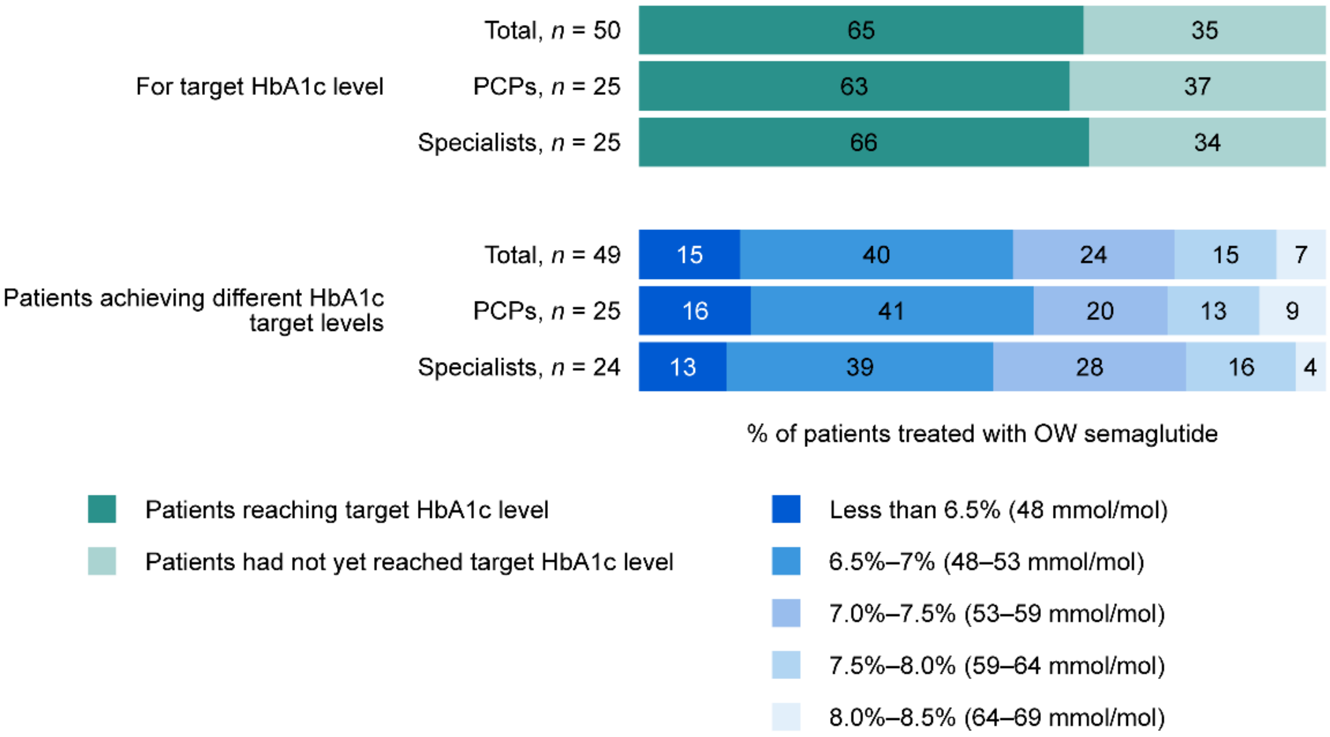

Percentage values may not total 100 due to rounding. Questions: (Top panel) What percentage of your patients using Ozempic® (semaglutide) have reached their target HbA1c level? (Bottom panel) Thinking about the patients who reached target HbA1c after initiation of Ozempic® (semaglutide), how were their target HbA1c levels distributed across the options listed below? 1) Less than 6.5% (48 mmol/mol); 2) 6.5%–7.0% (48–53 mmol/mol); 3) 7.0%–7.5% (53–59 mmol/mol); 4) 7.5%–8.0% (59–64 mmol/mol); 5) 8.0%–8.5% (64–69 mmol/mol).

HbA1c, glycated hemoglobin; *n*, number of participants; OW, once weekly; PCPs, primary care practitioners; T2D, type 2 diabetes.

Fig. S2: Proportions of patients with T2D who reached and did not reach their HbA1c target receiving 0.5 mg or 1.0 mg OW semaglutide, as reported by 50 prescribing physicians in Canada

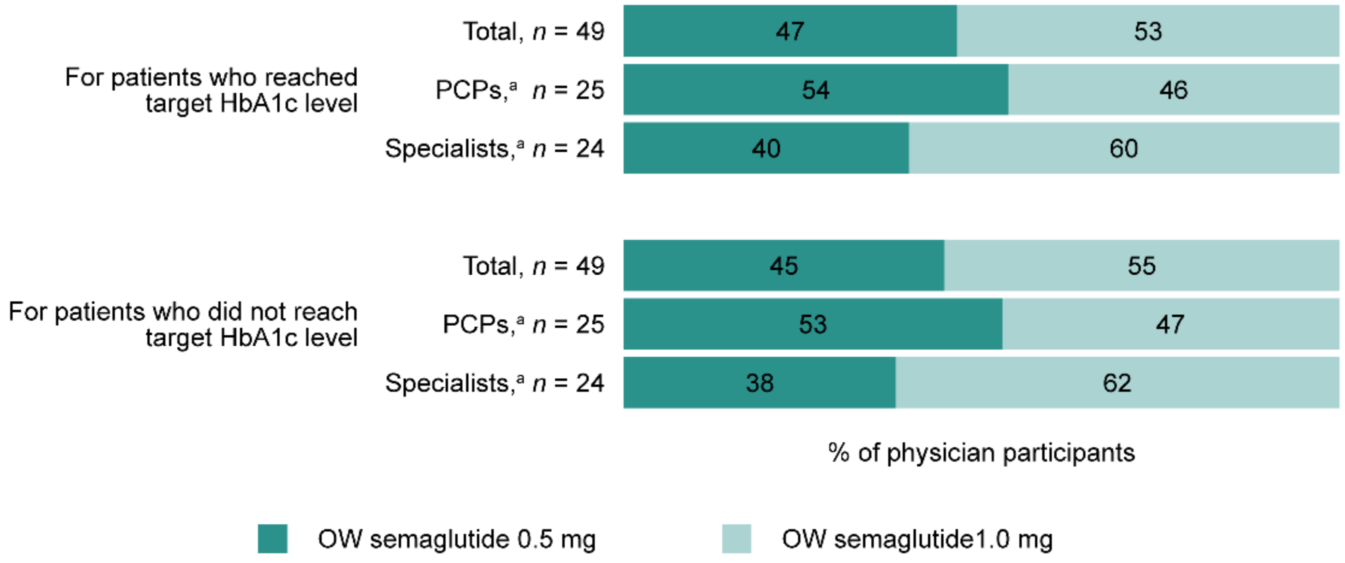

Questions: (Top panel) For patients who have reached target after initiation of Ozempic® (semaglutide), what proportion are on 0.5 mg maintenance dose versus 1.0 mg dose? (Bottom panel) For patients who have not yet reached target after initiation of Ozempic® (semaglutide), what proportion are on 0.5 mg maintenance dose versus 1.0 mg dose?
[a]Significant difference between PCPs and specialists at 90% confidence limit.

HbA1c, glycated hemoglobin; *n*, number of participants; OW, once-weekly; PCPs, primary care practitioners; T2D, type 2 diabetes.

Fig. S3: Physician-reported reasons for discontinuation of OW semaglutide[a] in patients with T2D in Canada

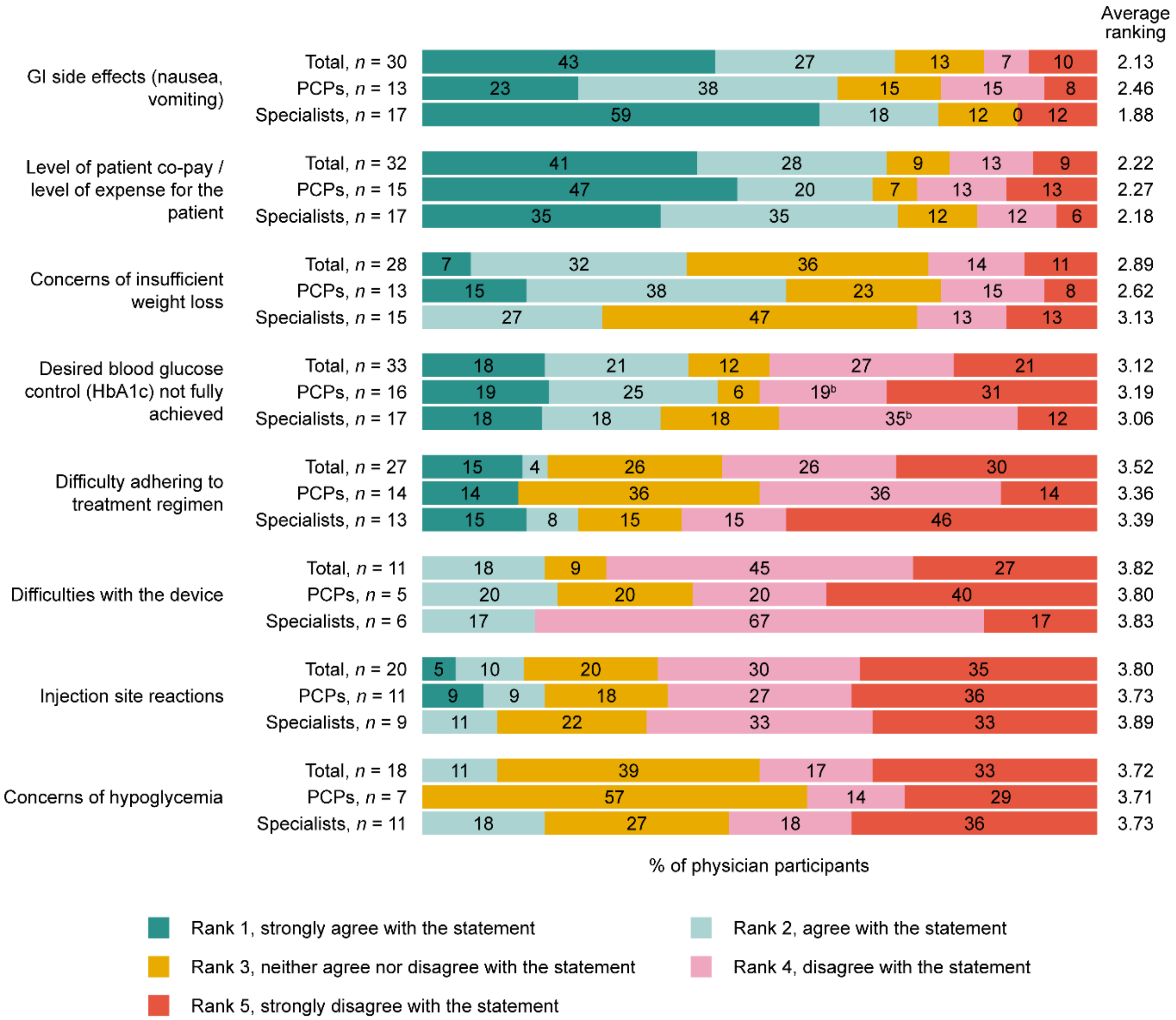

Percentage values may not total 100 due to rounding. Question: What do you believe are the main reasons for discontinuation of Ozempic® (semaglutide)?

[a]Other reasons for discontinuation OW semaglutide included cost of drug (other mentions).

[b]Significantly more specialists disagreed with the statement that desired blood glucose control not fully achieved was the main reason for discontinuation OW semaglutide, compared with PCPs at 90% confidence limit.

GI, gastrointestinal; HbA1c, glycated hemoglobin; *n*, number of participants; OW, once-weekly; PCPs, primary care practitioners; T2D, type 2 diabetes.

Fig. S4: Physician-reported patient concerns with OW semaglutide for T2D in Canada

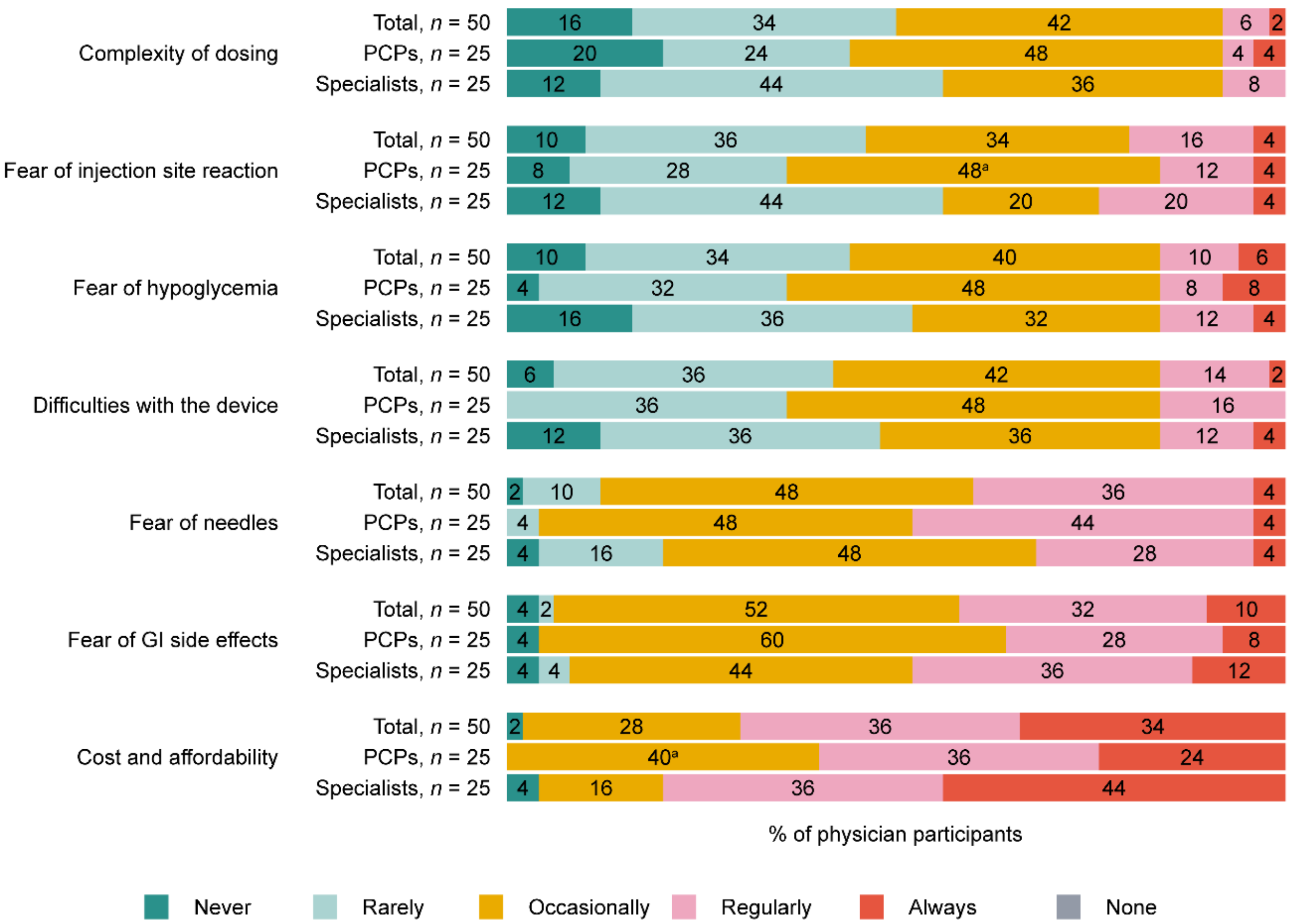

Question: How often are the following concerns expressed by your patients treated with Ozempic® (semaglutide)?
[a]Significant difference between PCPs and specialists at 90% confidence limit.

GI, gastrointestinal; *n*, number of participants; OW, once-weekly; PCPs, primary care practitioners; T2D, type 2 diabetes.

Ozempic® (semaglutide) Physician Understanding and Experience Survey

20 mins

Quota: Canada-50

Thank you for your interest in our market research study

Before passing on to the main survey we will first ask some screener questions to ensure that you can participate in this survey.

PERSONAL DATA

Please be informed that we limit the personal data we hold about you to contact details and information about your specialization in order to conduct market research.
Your responses and any personal contact information you provide in completing the survey will be:

i1. Processed by the IQVIA group of companies on a strictly need-to-know basis, for purposes of informing IQVIA and its client(s) of current and on-going trends in the management of Type 2 Diabetes Mellitus and for any follow-up contact that you have consented to

i2. Kept confidential, and not disclosed to the sponsoring pharmaceutical company or any third party outside of IQVIA except in aggregated or non-identified form, provided however that your identity may be disclosed to the sponsoring pharmaceutical company if you give your consent for your personal details to be passed on in the event of adverse event reporting, or if required by applicable law to meet mandatory regulatory reporting requirements

i3. Stored securely on IQVIA servers located in the EU and US in accordance with applicable data protection laws and retained only as long as necessary for the purposes of use outlined herein.

ADVERSE EVENTS REPORTING

We are now being asked to pass on to our client details of adverse events that are mentioned during the course of market research. Although what you say will, of course, be treated in confidence, should you raise during the discussion/survey an adverse event in a specific patient or in a specific number of patients, we will need to report this even if it has already been reported by you directly to the company or the regulatory authorities using the normal reporting processes.
In such a situation you will be contacted to ask whether or not you are willing to waive the confidentiality given to you under the market research codes of conduct specifically in relation to that adverse event/product complaint. All other information that you provide during the course of the survey will remain confidential.

In the event of an adverse event/side effect being found during the analysis of this research, are you willing to waive the confidentiality given to you under the Market Research Codes of Conduct specifically in relation to that adverse event? Please note that if you consent to a follow up of the Adverse Event, your name will not be linked in any way to your responses given during the survey, other than in relation to the adverse event.

☐ Yes
☐ No

CONFIDENTIALITY AGREEMENT: You acknowledge that in the course of this study, proprietary information regarding products and product development, and other trade secrets and know-how may be disclosed, and by participating in this study you agree to hold all such information confidential and to not disclose it to any third party or use it for any other purpose whatsoever. You also agree not to disclose any part of the following pages, which are proprietary material of IQVIA and its clients. You are required to accept the above confidentiality agreement in order to participate in this survey.
I confirm my agreement to proceed with this research and to the processing of my personal information provided to IQVIA, as stated above.

Please indicate whether or not you accept:

**Accept our conditions and start now**	**1**	
**Cancel**	**2**	**CLOSE**

Introduction

Thank you for taking the time to reflect and comment on your experiences of treating patients with Type 2 diabetes mellitus (T2DM) with semaglutide (Ozempic®). Semaglutide (Ozempic®) is a new glucagon-like peptide-1 (GLP-1) analogue which is administered subcutaneously once weekly for the treatment of adults with T2DM. It stimulates insulin and suppresses glucagon secretion in a glucose-dependent manner.

Please respond to the questions by ticking the answer that best reflects your experience. There are no right or wrong answers to any of these questions. Please answer the questions based on your memory; you are not required to go through individual patient records.

Screener

S1. Could you please confirm your specialty?

i1. General Practice	☐
i2. Family Medicine	☐
i3. Internal Medicine	☐
i4. Cardiologist	☐
i5. Endocrinologist	☐
i6. Diabetologist	☐
i7. Other specialty, please specify	☐

Single select

S2. On average, how many T2DM patients have you personally seen during the last 12 months?

Open end numeric

S3. Out of the total T2DM patients personally seen by you in the last 12 months, how many have you treated or are currently treating with Ozempic® (semaglutide)?

Open end numeric
If S3<=1, TERMINATE

S4. When did you first prescribe Ozempic® (semaglutide) to any of your patients?

i1. 1 month ago or less	☐
i2. 2 months ago	☐
i3. 3 months ago	☐
i4. 4 months ago	☐
i5. 5 months ago	☐
i6. 6 months ago	☐
i7. 7 months ago or more	☐

Single select
For cognitive debriefs: If i1/i2/i3 selected, TERMINATE
For the main survey: If i1/i2/i3/i4/i5 selected, TERMINATE

S5. When did you last prescribe Ozempic® (semaglutide) to any of your patients?

i1. Less than 14 days back	☐
i2. 15 days - 1 month	☐
i3. 1 – 2 months	☐
i4. 2– 3 months	☐
i5. 3– 4 months	☐
i6. 4– 5 months	☐
i7. 5–6 months	☐

Single select

Patient Caseload

Doctor, you mentioned that you have treated or are currently treating [insert S3] patients with Ozempic® (semaglutide)

1) How would you divide your T2DM patients being treated with Ozempic® (semaglutide) among each of the following treatment regimens?

	Number of patients
i1. Semaglutide monotherapy when metformin is not appropriate	
i2. Semaglutide in combination with one OAD	
i3. Semaglutide in combination with two OADs or more	
i4. Semaglutide in combination with insulin	
i5. Other[please specify____________________]	
	SUM=[insert S3]

Open end numeric
SUM[i1:i5] =S3

2) Prior to treatment with Ozempic® (semaglutide), what anti-diabetic treatment were these patients receiving? Please divide your Ozempic® (semaglutide) patients among the following treatment regimens.

	Number of patients
i1. One Oral Anti- Diabetic (OAD) medication	
i2. Two OADs	
i3. Three or more OADs	
i4. GLP-1 RA (+/- OAD)	
i5. Basal (+/- OAD)	
i6. Basal + Bolus (+/- OAD)	
i7. Basal + GLP-1 RA (+/- OAD)	
i8. Continuos Subcutaneous Insulin Infusion (CSII) / Insulin Pump	
i9. Treatment naïve	
i10. Other, [please specify____________________]	
	SUM=[insert S3]

Open end numeric
SUM[i1:i10] =S3

3) What do you believe are the main reasons for prescribing Ozempic® (semaglutide) to your patients with T2DM? Please rank the top 5 from the list below, where Rank 1 is the top reason.

	Rank (1-5)
i1. It offers superior glycemic control in T2DM patients	
i2. It has potential to reduce the risk of cardiovascular events	
i3. It delivers superior weight loss	
i4. It needs to be taken once a week potentially leading to better patient adherence	
i5. It offers value for money	
i6. Better device option	
i7. Other reasons, [please specify____________________]	

Rank 1-5 allowed
If i7 is ranked, then ask "Please specify the other reason for your patient not reaching the target level of HbA1c" /Open text

Patient Characteristics and Treatment Initiation

Doctor, the next section will focus on the characteristics of your Ozempic® patients. Many questions will be asked in proportion instead of patient numbers in the remainder of the survey.

4) What were the different types of patients for whom you have prescribed Ozempic® (semaglutide)? *(Please select all that apply)*

i1. Patients where there is a concern of inadequately controlled blood glucose (HbA1c)	☐
i2. Patients with risk of hypoglycaemia	☐
i3. Patients who found adherence to previous treatment difficult	☐
i4. Patients with problem of weight gain	☐
i5. Patients with established cardiovascular risk like previous stroke or myocardial infarction Other, please specify	☐
i6. Other, [please specify____________________]	☐

Multiple select

5) At the time of initiating Ozempic® (semaglutide) treatment, what were the key patient characteristics?
Display statement for the entire question and all four screens

a. What proportion of your Ozempic® (semaglutide) patients fall in the following age groups?

Age characteristics	% patients
i1. Age below 50 years	
i2. Age 50-65 years	
i3. Age above 65 years	
Total (100%):	100%

Numeric response
If total is ≠ 100% then alert "Please total up to 100%"

b. What proportion of your Ozempic® (semaglutide) patients fall in the following BMI ranges?

BMI	% patients
i1. Body Mass Index (BMI) below 30	
i2. Body Mass Index (BMI) 30-35	
i3. Body Mass Index (BMI) above 35	
i4. Unknown	
Total	100%

BMI or Body Mass index is defined as the body mass (kg) divided by the square of the body height (m^2)

Numeric response

If total is ≠ 100% then alert "Please total up to 100%"

c. What proportion of your patients showed the following HbA1c levels?

HbA1c levels	% patients
i1. HbA1c level below 8.0% (64 mmol/mol)	
i2. HbA1c level 8.0% to 8.5% (64 to 69 mmol/mol)	
i3. HbA1c level 8.6% to 9.0% (70 to 75 mmol/mol)	
i4. HbA1c level above 9.0% (75 mmol/mol)	
i5. Unknown	
Total	100%

Numeric response

If total is ≠ 100% then alert "Please total up to 100%"

If respondent inputs more than 0% for i2, i3 or i4 then display c1

c1. For patients with HbA1c levels of 8.0% or more, please indicate if there was a clinical consequence or not.

	With clinical consequence (e.g. hypo/hyper episode where patient was unable to treat him/herself)	Without clinical consequences
i1. HbA1c level 8.0% to 8.5% (64 to 69 mmol/mol)	☐	☐
i2. HbA1c level 8.6% to 9.0% (70 to 75 mmol/mol)	☐	☐
i3. HbA1c level above 9.0% (75 mmol/mol)	☐	☐

Single select for each row

Display c1 options basis data filled for corresponding entries in C

6) Thinking about your Ozempic® (semaglutide) patients, who initiated the conversation to switch to Ozempic® (semaglutide)? What proportion of your patients would fall in the following categories:

i1. Conversation was initiated by me	
i2. Conversation was initiated by the Patient	
i3. Conversation was initiated by others [please specify____________________]	
Total	100%

Numeric response

If total is ≠ 100% then alert "Please total up to 100%"

7) Was Ozempic® (semaglutide) prescribed at the same visit, when the conversation to include Ozempic® (semaglutide) was initiated? What proportion of your patients would fall in the following categories:

i1. Yes it was prescribed during the same visit	
i2. No it was prescribed during the next visit	
i3. No it was prescribed after 2-5 visits	
Total	100%

Numeric response
If total is ≠ 100% then alert "Please total up to 100%"

Treatment Response

In this section we will talk about the treatment response to Ozempic® (semaglutide) in your patients.

8) Is there a change in the frequency of patients' blood glucose self-monitoring after treatment with Ozempic® (semaglutide) compared to before Ozempic® (semaglutide) treatment?

i1. Blood glucose self-monitoring has decreased	☐
i2. Blood glucose self-monitoring has increased	☐
i3. No change in blood glucose self-monitoring	☐

9) What percentage of your patients using Ozempic® (semaglutide) have reached their target HbA1c level? Please total to 100% across the options listed below.

i1. Ozempic® patients reaching target HbA1c level	
i2. Ozempic® patients unable to reach target HbA1c level	
Total	100%

Numeric response
If total is ≠ 100% then alert "Please total up to 100%"

10) Thinking about your patients who reached target HbA1c after initiation of Ozempic® (semaglutide), how were their target HbA1c levels distributed across the options listed below? *(example: if target level was 6.5%-7% for 30% patients then fill 30% against i2)*

Target HbA1c Level	% T2DM Patients
i1. Less than 6.5% (48 mmol/mol)	
i2. 6.5% - 7% (48 to 53 mmol/mol)	
i3. 7.1% - 7.5% (54 to 59 mmol/mol)	
i4. 7.6% - 8.5% (60 to 69 mmol/mol)	
Total (100%):	100%

Numeric response
If total is ≠ 100% then alert "Please total up to 100%"

11) Thinking about your patients who have reached target HbA1c after initiation of Ozempic® (semaglutide), on average how many visits and how long did it take for them to reach their target level of HbA1c?
(For time duration, please answer the question in either months OR weeks OR days OR a combination of the three)

i1. Number of visits (average)	___

i2. Time duration (average)	□□□□□□□□__months<br>□□□□□□□□__weeks<br>□□□□□□□□__days

i1- Numeric responses, 2 digits
i2- Single select between Months/weeks/days and then allow Numeric response, 2 digit

12) For patients who have reached target after initiation of Ozempic® (semaglutide), what proportion are on 0.5 mg maintenance dose versus 1.0 mg dose?

Maintenance dose for Glycemic control	% T2DM Patients
i1. 0.5 mg	
i2. 1.0 mg	
i3. Other dose[please specify____________________]	
Total (100%):	100%

Numeric response
If total is ≠ 100% then alert "Please total up to 100%"
If i3 > 0, then ask "Please specify the other dose" / Open text

13) For patients who have not reached target after initiation of Ozempic® (semaglutide), what proportion are on 0.5 mg maintenance dose versus 1.0 mg dose?

Maintenance dose for Glycemic control	% T2DM Patients
i1. 0.5 mg	
i2. 1.0 mg	
i3. Other dose[please specify____________________]	
Total (100%):	100%

Numeric response
If total is ≠ 100% then alert "Please total up to 100%"
If i3 > 0, then ask "Please specify the other dose" / Open text

14) Among the patients who have not reached their target HbA1c levels after initiation of Ozempic® (semaglutide), what do you believe are the reasons for not reaching target levels? Please rank the top 5 from the list below, where Rank 1 is the top reason.

	Rank (1-5)
i1. Patient has recently initiated the treatment	
i2. Lack of adherence	
i3. Fear of reaction at administration site	
i4. Fear of hypoglycaemia	
i5. GI side effects (nausea, vomiting)	
i6. Difficulties with dose adjustment	
i7. Difficulties with the device	
i8. Other reasons, [please specify____________________]	

Rank 1-5 allowed
If i8 is ranked, then ask "Please specify the other reason for your patient not reaching the target level of HbA1c" / Open text

15) What do you believe are the main reasons for discontinuation of Ozempic® (semaglutide)? Please rank the top 5 from the list below, where Rank 1 is the top reason.

	Rank (1-5)
i1. Desired blood glucose control (HbA1c) not fully achieved	
i2. Concerns of weight loss	
i3. Concerns of hypoglycaemia	
i4. Level of patient co-pay/ level of expense for the patient	
i5. Difficulty in adhering to treatment regimen	
i6. Injection site reactions	
i7. GI side effects (nausea, vomiting)	
i8. Difficulties with the device Ozempic® (semaglutide)	
i9. Other reasons, [please specify___________________]	

Rank 1-5 allowed
If i9 is ranked, then ask "Please specify the other reason for your patient not reaching the target level of HbA1c" /Open text

16) How often are the following **concerns** expressed by your patients treated with Ozempic® (semaglutide)?

	(1) Never	(2) Rarely	(3) Occasionally	(4) Regularly	(5) Always
i1. Fear of injection site reaction					
i2. Fear of GI side effects					
i3. Fear of hypoglycaemia					
i4. Difficulties with the device					
i5. Fear of needles					
i6. Complexity of Dosing					
i7. Other concerns, [please specify___________________]					

*Should you fill in 'Other' as one of your answers, you will have a chance to specify that answer on the next screen.
Single response per row
If i7 > 0 then ask "Please specify the other concern expressed by your patients"
Open text

Confidence level

17) Please compare your **SATISFACTION** of treating patients with Ozempic® (semaglutide) **compared to other GLP-1 receptor agonists (RAs)** based on each of the following:

	(5) Much more satisfied with Ozempic®	(4) More satisfied with Ozempic®	(3) Equally satisfied with both	(2) More satisfied with **other GLP-1 Ras**	(1) Much more satisfied with **other GLP-1 RAs**
i1. Reaching HbA1c target					
i2. Weight management					
i3. Incidence of hypoglycaemia					
i4. Flexibility of dosing					
i5. Ease of training the patients					
i6. Overall side effect profile					
i7. Cost effectiveness					
i8. Simplicity of therapy					
i9. Patient adherence					
i10. Number of injections					
i11. Patient satisfaction					

Single response per row

18) Based on your experience, how **motivated** are patients to reach their target blood glucose levels with Ozempic® (semaglutide) **compared to other GLP-1 RAs**?

(5) Ozempic® has much more potential to improve motivation	(4) Ozempic® has slightly more potential to improve motivation	(3) Ozempic® is somewhat perceived to be the same as **other GLP-1 RAs**	(2) **other GLP-1 RAs** have slightly more potential to improve motivation	(1) **other GLP-1 RAs** have much more potential to improve motivation

Single response only

19) Based on your experience, what are your **concerns** for treating patients with Ozempic® (semaglutide) **compared with other GLP-1 RAs**?

	(5) Much more concerned with **other GLP-1 RAs**	(4) More concerned with **other GLP-1 RAs**	(3) Equally concerned with **both**	(2) More concerned with Ozempic®	(1) Much more concerned with Ozempic®
i1. Concern of patient struggling with nausea and other GI side effects					
i2. Concern of patients gaining weight					
i3. Concern of patients having hypoglycaemia					

i4. Other concerns, [please specify________________]					

*Should you fill in ‘Other’ as one of your answers, you will have a chance to specify that answer on the next screen.
Single response per row
If i4 > 0 then ask “Please specify the other concern”
Open text

Thank you for participating in this research study.

IQVIA may wish to re-contact you after the interview to clarify any follow-up queries related to this study. Please confirm if you agree to be re-contacted on this basis.

☐ I consent to be re-contacted for follow-up queries on this study

☐ I do not wish to be re-contacted for follow-up queries on this study
